# Executive Function Capacities, Negative Driving Behavior and Crashes in Young Drivers

**DOI:** 10.3390/ijerph14111314

**Published:** 2017-10-28

**Authors:** Elizabeth A. Walshe, Chelsea Ward McIntosh, Daniel Romer, Flaura K. Winston

**Affiliations:** 1 Annenberg Public Policy Center, University of Pennsylvania, Philadelphia, PA 19104, USA; dan.romer@appc.upenn.edu; 2 Center for Injury Research and Prevention, Children’s Hospital of Philadelphia, Philadelphia, PA 19104, USA; WARDC8@email.chop.edu (C.W.M.); WINSTON@email.chop.edu (F.K.W.)

**Keywords:** executive function, cognitive control, adolescents, young drivers, driving behavior, motor vehicle crashes

## Abstract

Motor vehicle crashes remain a leading cause of injury and death in adolescents, with teen drivers three times more likely to be in a fatal crash when compared to adults. One potential contributing risk factor is the ongoing development of executive functioning with maturation of the frontal lobe through adolescence and into early adulthood. Atypical development resulting in poor or impaired executive functioning (as in Attention-Deficit/Hyperactivity Disorder) has been associated with risky driving and crash outcomes. However, executive function broadly encompasses a number of capacities and domains (e.g., working memory, inhibition, set-shifting). In this review, we examine the role of various executive function sub-processes in adolescent driver behavior and crash rates. We summarize the state of methods for measuring executive control and driving outcomes and highlight the great heterogeneity in tools with seemingly contradictory findings. Lastly, we offer some suggestions for improved methods and practical ways to compensate for the effects of poor executive function (such as in-vehicle assisted driving devices). Given the key role that executive function plays in safe driving, this review points to an urgent need for systematic research to inform development of more effective training and interventions for safe driving among adolescents.

## 1. Introduction

Motor vehicle collisions (MVC) remain the leading cause of death and injury in adolescents worldwide [1,2]. In the US and Canada, MVCs account for a third of all adolescent deaths and over 259,000 injuries [1,3]. Young drivers also represent a risk to their passengers and other road users: young drivers are involved in a disproportionate amount of crashes, with a fatal crash rate three times higher than that of middle-aged drivers, accounting for vehicle miles traveled [4]. Prior studies have shown that teen driver crashes are attributed to a greater number of decision errors (e.g., inappropriate speed for conditions), risky driving behaviors, inadequate surveillance and hazard perception, distracted driving (e.g., cell phone use) and the presence of peers [4,5,6,7].

While inexperience likely plays a part, many of these crash-contributing factors imply poor executive function. Higher-level executive function (EF; also called executive control) underpins the ability to manage complex tasks, such as driving, by facilitating continued focus on the task or goal (e.g., attention on the road, driving tasks and the destination), while filtering relevant and irrelevant information (e.g., dealing with distractions) and adapting to the task demands (e.g., navigating roadway conditions and traffic). EF has been shown to develop through adolescence and into early adulthood with maturation of the frontal lobe [8,9,10]. Furthermore, low EF is related to impulsivity, sensation seeking and other risk-taking behaviors in teens [11,12,13]. Limited EF due to atypical development may contribute to poor attention and decision making, impulsivity, and the risky driving behaviors that contribute to the elevated crash risk among teen drivers [14,15,16,17]. However, sensation seeking and rationalized risk-taking (i.e., weighing risks and benefits rather than impulsive risk-taking) also appear to increase with EF ability in adolescence (see the Life-span Wisdom Model [13]), indicating a complex relationship between EF and adolescent risk-taking behavior.

Observational and experimental studies support the role of EF ability in adolescent crash risk. Findings point to increased crash risk during distracted driving, greater frontal lobe activation during simulated driving, and poorer driving performance in older adults and clinical samples with cognitive impairments [14,18,19,20,21]. For example, adolescent drivers with EF impairments—as in Attention-Deficit/Hyperactivity Disorder (ADHD) and Autism Spectrum Disorder (ASD)—have a higher crash and injury risk, according to some reports [22,23]. These conditions are characterized by increased difficulty in paying attention, coordination, organized planning and making decisions while driving, as well as by making more driving errors (particularly when there is increased demand on EF resources) [23,24,25,26]. In fact, Garner et al. [27] discerned that inattention, rather than impulsivity, better predicts risky driving in young drivers with ADHD. However, more recent studies of adult drivers with ASD reveal mixed findings of selective effects, whereby these drivers display more lapses, errors and slower reaction times [28,29,30], but also exhibit better rule-following for checking cross-traffic and the use of indicators [28].

However, the specific role of EF constructs in driving performance has not been examined systematically in adolescent drivers [31]. In order to improve teen driver safety and reduce injury and death on the roads, it is crucial that we better understand which discrete domains and sub-processes of EF are key to safe driving so that training and other countermeasures can be geared towards addressing the root causes of adolescent crashes. This review examines EF capacities in adolescents and how they relate to driving performance and outcomes (i.e., crashes). This review also aims to assess the state-of-the-art tools in measurement and methods regarding EF and adolescent driving, to suggest future directions for research, and to offer some guidance for training and intervention development. First, we begin with a brief conceptual outline of the nature of executive function.

## 2. Executive Function

The umbrella term “executive function” is used to describe a number of top-down control processes that allow us to regulate our thoughts and behavior by managing incoming sensory information, directing attention allocation and selecting behavioral responses see [9,32]. These EF processes are often associated with the prefrontal cortex of the brain, and map onto a number of related but separable domains or constructs that operate with limited resource capacities [9,33]. Three factor constructs of EF are commonly reported: working memory, inhibition (response inhibition and interference control) and set-shifting [8,9,33].

Working memory is the capacity for information monitoring, updating and manipulation in the moment (including monitoring multiple tasks) that pertains to visual, verbal and spatial information [9,34,35]. Inhibitory control is the capacity to filter, suppress or resist irrelevant or distracting sensory information or primary reflexes. Specifically, interference control (or selective attention) allows distractors to be ignored, while response inhibition allows for voluntarily suppression of prepotent behavioral responses, and appropriate response execution [9,33,36]. Set-shifting (cognitive flexibility or task switching) is the ability to adapt to changing goals or task demands, or to shift mental set/perspective [9,33]. Other components such as such as forward planning, prospective memory, and attention (vigilance/sustained attention and visuospatial orienting) have also been reported, but to a lesser extent [9].

In the task of driving, drivers are required to maintain focus on the road (i.e., sustained attention) and goal/destination (using working memory), while constantly adapting speed to the demands of the situation (flexibility/set-shifting) and managing distraction from peers, in-car technology or other sources (i.e., inhibiting responses to distraction and/or using working memory to multitask). These key EF constructs have been shown to be distinct, but highly interrelated, and can depend upon each other [9]. For example, good inhibitory control helps maintain focus on the road, and keeping the goal and task in mind helps avoid distraction. These construct domains and sub-processes of EF also have a shared neural basis within the prefrontal cortex (mainly dorsolateral) and anterior cingulate, and the associated neural networks, highlighting their interrelatedness [9,33,37].

Within the driving literature reviewed here, EF was most commonly defined as a broad set of higher-level cognitive processes that play a supervisory or managerial role in complex behaviors, often with reference to Miyake’s model of EF as both unified and diverse [33]. Within this definition, the three core components of EF were prominent, with some studies naming as little as two EF sub-processes—such as inhibition and/or working memory capacity [38]—where others provided an extensive list of EF sub-processes [39]. Typical development of EF progresses rapidly during childhood and adolescence, alongside structural changes in frontal grey and white matter tracts that strengthen brain networks, but some EF capacities don’t reach full maturation until later into the 20s [40,41,42]. Response inhibition and set-shifting have been shown to mature by mid-adolescence (by ~age 12–15 years), but working memory and sometimes interference inhibition have been evidenced to mature later in early adulthood [8,9]. While infants can update the contents of their working memory, the capacity is very limited: the ability to maintain and manipulate many pieces of information develops slowly and much later during adolescence, along with interference inhibition [42,43]. Thus, the development of EF processes is not homogenous and the length of the trajectories are not fixed. Furthermore, much of the variability in executive capacities can be attributed to individual differences, whereby some late adolescents can perform better than the adult average [44].

## 3. Methods

A literature search was conducted to identify relevant recent studies of EF capacity in adolescents that relate to driving outcomes. The aim was to identify the relative role of the different EF capacities and sub-processes that are important for driving performance in young drivers. The search was undertaken using Science Direct and PubMed databases to identify all relevant papers published between 2000 and July 2017. A combination of search terms was used, such as “executive”, “driving”, “adolescent”, and “young driver”. Studies were included if they related EF capacity to driving in a sample of typically developing adolescent drivers, or driver samples with a mean age between 16–21. Studies that analyzed atypically developing young drivers (e.g., ADHD, ASD), or middle-aged and older adults, were not included in this review. The reference lists of all included studies were searched for additional relevant studies.

## 4. Results

Firstly, we provide an overview of the methods used, then delineate the role of global EF capacity in adolescent driving, followed by a review of the findings that link different EF capacities and sub-processes to driving outcomes in young drivers. Table 1 presents the recent studies (from 2000 to 2017) that investigated the contribution of EF capacities to negative driving behavior and increased crash risk among adolescent drivers. A range of methodologies were used, precluding replication or meta-analysis, but the table is instructive in presenting the range of subjective and objective measures of EF used as well as the range of methods used for measuring driving outcomes, both subjective self-report scales (e.g., the Driver Behavior Questionnaire: DBQ), and objective performance-based measures of simulated driving.

For measuring EF, the majority of studies used neuropsychological performance-based tasks (e.g., Go/No-Go task of response inhibition). Some studies also examined resource sharing between EF domains and driving by taxing drivers with a secondary EF task, and observing deficits in driving performance. Fewer studies employed self-report measures of real-world EF behavior (namely the clinical Behavior Rating Inventory of Executive Function: BRIEF) [31,14,16,45], and only one paper reviewed here utilized both subjective and objective measures of EF [31]. These questionnaires consisted of a number of subscales targeting specific EF sub-processes, but a total global EF score was utilized most often in the analyses. Poor global EF as measured on the BRIEF predicted more lapses, errors and violations reported on the DBQ, independently of age and sex [45]. Low global EF was also associated with increased engagement in distracted driving—including texting while driving, specifically [14,16]. In addition, self-reported cognitive failures (i.e., absent-mindedness), which represent a failure of EF, and poor vigilance (but not executive attention) were also related to distracted driving as measured by lapses on the DBQ [46]. However, it is important to discern the differential role of individual executive domains and sub processes, as not all of them may relate to all negative driving outcomes [31]. The following review consists mainly of studies examining the three core domains of EF as outlined by Miyake et al. [33]. However, measures of attention were also included in some studies, and so we have included a brief section to highlight these findings also.

### 4.1. Working Memory

Most of the studies reviewed here found a significant relationship between poor working memory and negative driving outcomes. Using self-reported driving outcomes, Pope et al. [31] found that poor self-reported WM (Working Memory) on the BRIEF was related to more incidents of MVCs, being pulled over and receiving traffic tickets, but only significantly increased the odds of being pulled over. Other studies using a simulated driving task as the driving outcome also found a relationship between WM and poorer driving performance. Ross et al. found that poorer verbal and visuo-spatial WM predicted more variation in lane position (swerving) [38], but not collisions or hazard responses [47]. Lane maintenance has also been shown to deteriorate when resources are taxed with a secondary WM task [38,47,48], but young drivers with high WM capacity at baseline were less influenced by the additional load [38]. Furthermore, a study by Mäntylä et al. [49] that compared composite scores across the three core EF domains clearly showed that poor WM performance alone (and not inhibition or set-shifting) predicted worse performance on the simulated lane change task in teen drivers.

Two studies contradict the hypothesis that better WM contributes to safer driving. Using self-reported outcomes, Starkey and Isler [39] reported that higher WM ability related to more self-reported risky driving and more accepting attitudes to risk among male teen drivers. Using simulated driving outcomes, Ross et al. [47] also found that higher visuospatial WM performance contributed to running more red and yellow traffic lights and shorter following distances in a simulated task.

### 4.2. Inhibition

In order to study the role of inhibition on negative driving outcomes, several investigators used retrospective study designs which compared those who had driving infractions to controls that did not. Two such studies found a relationship between inhibition (self-reported and quantitatively measured) and traffic violations. Lower self-reported inhibition on the BRIEF has been significantly associated with the increased odds of being pulled over and receiving a ticket in 16–19 year olds [31]. One UK study [37] compared a group of young drivers recruited from a speed awareness course (“offenders”) to a group of age-matched controls on two performance-based measures of response inhibition (Stop-Signal and Go/No-Go tasks). The young offenders had poorer inhibitory control (on the Go/No-Go task alone), with faster reaction times overall suggesting a speed-accuracy trade off. Hatfield et al. [50] found that poorer inhibitory control on the Go/No-Go task positively correlated with total “unsafe” driving (pre-defined responses to hazards/risk events) and speeding in a simulated driving task (but not lane maintenance). In contrast, poor response inhibition on the Stop-Signal task (SST) has been associated with poor lane maintenance, but not with other measures of speeding or with running red lights [46]. In addition, drivers with poorer SST performance showed increased speeding in the presence of peer passengers (but no difference in lane position, running traffic lights, collisions, braking or deceleration for road hazards) [51], with better Go/No-Go performance related to less red light running in the presence of cautious peers [52].

Three contradictory studies found no association between inhibitory control and risky driving using both simulated and self-report measures [39,49,53]. However, when comparing across domains, Ross et al. [47] found that poor SST (but not Go/No-Go) performance and poor WM contributed to more variation in lane position, and that poor inhibitory control alone (on both the SST and Go/No-Go) contributed to slower hazard responses and more crashes on a simulated task. Additionally, Guinosso et al. [48] reported that higher Stroop inhibitory control (and higher intelligence quotient [IQ]) predicted better simulated driving performances in late adolescents, whereas attention and flexibility/set-shifting did not.

### 4.3. Set-Shifting

Few studies examined set-shifting (or flexibility), but of those that did, none found a significant relationship with driving in adolescence. Set-shifting was not related to self-reported crashes or citations [31], or with simulated risky driving, speed or lane maintenance [48,49,53].

### 4.4. Attention

Among the studies reviewed above, a small number also examined the role of sustained attention/vigilance in adolescent driving. Sustained attention is a lower level attention capacity to maintain focus, which encompasses filtering information and ignoring distractors and irrelevant or conflicting sensory input, and thus overlaps with the EF processes defined above (particularly inhibition and working memory) [9]. Poorer vigilance/sustained attention was significantly related to more self-reported attentional lapses on the DBQ [47]. However, a factor of sustained attention (and forward planning) was not found to predict risky driving when compared to inhibition and WM [39]. In addition, Guinosso et al. [48] found that general attention (with more focus on alerting) was associated with, but did not predict, simulated lane maintenance in late adolescent drivers. Often the definitions of attention overlap with those of the EF domains, and as this review focused on the commonly reported core constructs of EF, studies of attention were not the focus of the literature search. However, the role of attention in driving and crash risk is apparent in the devastating effects of distracted driving behavior reviewed elsewhere [54].

## 5. Discussion

The general role of EF in driving is quite clear, whereby young drivers with inefficient EF make more errors, engage in more dangerous driving behaviors and are at a higher risk of crashing. However, this review attempted to tease apart the role of the different EF constructs and sub-processes, and how they differentially relate to driver risk. Despite some mixed findings, it appears that poor working memory, and to some extent inhibition, most consistently related to negative driving outcomes in adolescents and young adults. In summary, teen drivers with low self-reported WM ability report more crashes and traffic citations. In addition, young drivers with low performance-based WM also self-report more inattentive driving (but not errors or violations), and exhibit poor lane maintenance, hazard detection and perception during simulated driving. A low WM capacity would indicate poorer ability to update information in the moment and manage the many subtasks of driving, plus additional secondary tasks that are common in real-world driving (talking with peers, listening to the radio, eating, drinking, and cell-phone use). Thus, it is easy to see the importance of WM in driving safely, particularly among learner or new drivers who have not yet automated many of the subtasks of driving (e.g., checking mirrors, moving through gears).

However, higher WM capacity can also lead to increased risk-taking while driving in young drivers (e.g., running traffic lights). This increased risk-taking while driving may be related to greater sensation-seeking that has been associated with WM development, whereby teens with higher WM capacity may feel more capable of managing the task demands and thus take more calculated or rationalized risks while driving [13,47]. This is consistent with the observed trend that violations increase while errors decrease with age among young drivers [55], indicating voluntary risk-taking and improved control of the driving task with experience.

Poor inhibition is sometimes associated with unsafe or risky driving, speeding and poor lane maintenance in a simulated task, but this depends on the measure of inhibitory control. Two response inhibition measures were most commonly used (Go/No-Go and SST), with less examination of interference inhibition (Stroop). In addition, drivers with a record of speeding offences display poorer response inhibition control compared to age-matched peers. These findings suggest that young drivers may find it difficult to inhibit impulses for speed, perhaps related to sensation-seeking, and to ignore distracting input which may contribute to the poor speed control and lane maintenance exhibited above.

The more comprehensive studies that used regression models rather than correlation coefficients, and that employed multiple measures to compare across domains, largely supported the conclusion that WM and inhibition in particular play a key role in adolescent driving outcomes [39,47,48,49]. This implies that teen drivers may not be equipped to ignore distractions, and may be particularly vulnerable in complex/demanding driving situations that require efficient monitoring and updating of information. Taken together, poor working memory and inhibition may both contribute to distracted driving behaviors and their negative consequences, which are highly prevalent among teens and young drivers [14,54]. Furthermore, poor working memory and inhibition related to negative driving outcomes in healthy teens may be exacerbated in young drivers with developmental disorders such as ADHD and ASD, accounting for the increased risk in these populations.

Studies examining set-shifting indicate that this domain of EF does not relate to negative driving outcomes in young drivers. This may indicate that the ability to task-switch or shift mental set may not be important for driving. However, this seems unlikely as real-world driving frequently calls for adaptive behavior and task switching. Alternatively, these results may be due to set-shifting maturing earlier, around the age of 15 years [8], leading to less variability among young drivers aged 16 and above. However, we are cognizant of the fact that the literature above mostly targeted WM and inhibition, with far fewer studies examining the role of executive attention and set-shifting, which may account for these findings. Furthermore, other EF capacities such as forward planning and prospective memory (which are also suggested to continue to develop across adolescence with frontal lobe maturation) may also play a role in driving performance, but have not been consistently examined in prior literature [56].

While the capacity to sustain attention on the task of driving likely plays a role in safe driving and crash-avoidance—as indicated by the few studies using tasks of attention above—this review did not focus on the levels of attention. Given that many definitions and tasks of higher-levels of attention overlap with the domains of EF [9], attention likely plays a role in the effects of each of the EF domains on driving outcomes as outlined above. While not included here, many of the reviewed papers also investigated the additional factors of impulsivity, IQ and risk propensity as additional contributing factors to adolescent driver risk [16,38,39,48,50]. Collectively, these findings are consistent with the hypothesis that frontal maturation and underdeveloped executive control may contribute to risk-taking behavior and crashes in adolescent drivers [11,12,13]. Thus, we should be considerate of the cognitive state, as well as the traits, of the adolescent driver when investigating risk [15]. In addition, individual differences and the social and emotional context may also be important predictors of risk on the roads [52].

### 5.1. Methodological Issues

A number of issues were apparent in the reviewed literature that reveal key gaps in the scientific foundation for the role of EF on adolescent driving performance, and may limit the weight of our conclusions. Foremost, the heterogeneous terminology and methodology make it difficult to compare across studies, limit generalizability, and may account for the somewhat mixed findings above. As EF refers to a collection of processes and constructs, it can be given any number of conceptual and operational definitions. Varying construct definitions (e.g., WM maintenance or updating) and different measures of the same construct (e.g., Stroop for interference inhibition vs Go/No-Go task measuring response inhibition) have revealed contrasting results, even within the same sample, which may be due to different underlying neural processing [37,47,49]. Mäntylä et al. [49] also highlighted the questionable construct validity of some commonly used neuropsychological measures of EF, such as the Wisconsin Card Sorting Task of set-shifting. Some tasks may not target a single underlying construct, but rather tax a number of overlapping EF capacities. Furthermore, it is difficult to compare findings between studies utilizing self-reported rating scales versus performance based measures of EF as they may be measuring different things [14]. In order to circumvent this issue, future studies should use both forms of measurement, include multiple well-characterized objective measures of distinct EF domains to account for task-specific features, and compare across constructs to discern the discrete relationships between components of EF and driving. The selection of objective EF tasks should consider findings from the neuropsychological literature that relate behavioral performances to distinct neural networks and activity in the frontal lobe.

The issue of methodological variability was also apparent in the measurement of the driving outcomes, where it is difficult to compare self-report to simulated driving. Even within studies of simulation, the specific drivers and outcome measures varied widely, as did the levels of simulation. Some studies used low fidelity driving simulators that did not require all performance indices (steering direction and response time, as well as braking), with limited driving outcome variables. For example, Mäntylä et al. [49] used a driving simulation that only required steering input (not braking or response times) and only measured lane maintenance, without events to measure risky-driving. More complex driving events that have potential for risk-taking (e.g., intersections for red-light running) may relate to other aspects of EF. Thus, it is not only difficult to generalize across varied simulated driving tasks in the studies reviewed here, but also difficult to generalize to real-world driving that is complex with a high potential for risky events. However, future studies should continue to use simulated driving tasks as they offer a unique opportunity to observe ecologically valid risk scenarios and contexts for adolescent drivers, which are important outcome measures to be included. Critically, we also observed that some studies did not control for the potentially confounding variables of age and sex [31,46], and two studies only included males in the sample [39,52] who have been shown to crash more than females [57]. Despite these methodological issues, we conclude that working memory and inhibition play a key role in the disproportionately high crash risk for teen drivers.

In addition, some of the limitations of the observational experiments above could be overcome by using additional methodological approaches, such as computational modeling. Computational modeling can be used as a powerful tool to explore the factors and parameters of complex driving behavior, and these models can be based on driving data, cognitive structure/architecture and conceptual models of how driver behaviors impact road safety [58,59,60].

### 5.2. Future Directions

More systematic research is needed to compare across domains of executive function and to discern the relationship between EF, personality traits and the social and emotional context. Future research should also examine individual differences in EF capacity, particularly during adolescent development, which may impact the capacity to drive safely. Furthermore, a longitudinal study of the development of EF and driving skill acquisition across adolescence could also help clarify the interaction between these two capacities, and how they relate to the disproportionate crash risk of adolescent drivers. For each of these endeavors, the addition of neuroscientific measures to examine information processing (temporally with an electro- or magnetic electro-encephalogram) and cognitive load (using functional near-infrared spectroscopy) in the frontal lobe would greatly advance the current understanding of the relationship between brain and driving behavior.

Despite the need for more research, we consider some potential avenues for interventions or solutions for reducing adolescent injury and mortality on the roads, in light of this review. First we consider how some readily available interventions can be improved. The Graduated Driver Licensing (GDL) program is one such intervention already in place that has had some success in reducing fatal crashes in novice teen drivers [61]. Currently, all 50 states have some form of GDL program which provides longer licensing times or more hours of behind-the-wheel experience. However, this may not be sufficient for enhancing the advanced skills needed for safe driving in complex situations, and may not be of much benefit in later adolescence (aged 17+ years [62]). This may be due to overly generalized training within the GDL, and we suggest that more tailored skills training that is targeted towards improving driving tasks related to the working memory and inhibitory control capacity could promote safer driving behavior. For example, training could be tailored to the differential developmental trajectories of the EF processes across adolescence and young adulthood, with an aim to improve driving skills that may be impacted by limited EF development.

Furthermore, screening measures could be developed and utilized to assess individual differences in EF capacity [38,63] and how these relate to potential risk on the road. For example, working memory screening tools are commonly used to assess fitness-to-drive in older adults [64], and could be adapted to screen for heightened risk in teens with ongoing WM development or atypical development. This screening could suggest when additional driver training is needed, and also inform personalized driver skill training to be completed during driver education. Training on a driving simulator could be a fruitful avenue for implementing individually-tailored training that focuses on any limited EF capacities in young drivers. Simulated driver training can expose adolescents to the driving task without the real-world risk, and this training could target performance/compensation for known impairments. For example, young drivers with poorer working memory performance could be trained to drive under complex circumstances with multiple directions/goals, or while multitasking. In addition, young drivers with limited inhibitory control could be trained to drive with exposure to distractors (which are ever present in the real-world). In fact, driving simulation is already being considered as a novel tool for assessment, intervention and training in both typically developing teens and those with clinical impairment [65]. In addition, gaming activity and skills have been associated with both better working memory capacity and driving performance in typically developing adolescents [49,66], which could open up a new avenue for intervention via more accessible computer game simulated driver training for high-risk adolescents.

Furthermore, cognitive researchers continue to explore new forms of EF training for improving academic performance, which could be examined for potential transfer to improved driving performance in teens. In fact, the success of EF training has been most pronounced in those with poorer performance [67], so it could benefit teens with lower levels or later maturation of EF capacities (including teens with ADHD and ASD). However, the evidence for EF training transfer to real-world functions is still mixed [67]. In older adults, there are mixed findings of cognitive training transfer to non-trained simulated driving tasks. Cassavaugh and Kramer found some modest evidence for transfer of computer-based training of attention, visuospatial working memory and manual control to simulated driving performance gains [68]. However, Cuenen et al. [69] found only marginally significant effects, with limited transfer to the driving task. Further investigation and replication is needed. Furthermore, ageing cognitive decline is not necessarily comparable to EF development during adolescence, and so this approach should be investigated in adolescents.

Ongoing technological advances in the development of autonomous vehicles has the potential to provide safer driving environments, particularly for those with limited EF capacities (including drivers with clinical impairments and developing adolescent drivers). Furthermore, with more knowledge of how EF capacities predict driving behavior in young drivers and clinical samples, we may begin to harness computational models for practically recognizing and predicting driver behavior, impairment and distraction in the moment (at a trait and state level). However, until the knowledge base and this vehicle technology is readily available, we must look to more immediately available solutions. Vehicle manufacturers are currently equipping vehicles with advanced driver-assistance systems that provide alerts or perform automatic functions in order to avoid crashes see [70]. For example, lane departure warnings have recently been shown to be effective in preventing crashes [71], and could be particularly useful in counteracting the high lane position variability evident in teen drivers in the simulated studies reviewed above. Further development of such systems should target the known limitations of teen driver capacities in order to help ameliorate the catastrophic outcomes of some common driving errors. In order to mitigate the dangers of distraction in teen drivers, we should look to technologies such as cell phone-blocking applications, which have already been evidenced as effective in teen drivers [72]. However, while these technologies are successful for isolated behaviors, we should also consider more holistic approaches and longer-term solutions for reducing risky driving behaviors and improving young driver safety.

## 6. Conclusions

In conclusion, from the existing literature, it seems that limited abilities to inhibit distracting information, and to monitor, update and integrate task-relevant information in the moment, contribute to poorer driving performance and higher crash-risk in adolescents. However, there has been insufficient examination of set-shifting, attention and other capacities such as planning and prospective memory, to rule out their involvement in driving performance. Thus, there is an urgent need for systematic research to inform the development of more effective training and interventions for safe driving among adolescents. Further rigorous examination of the relationship between different EFs and driving in young drivers that is supplemented with alternative methodological approaches (neuroscientific measures and computational modeling), would greatly advance current knowledge.

Driver education and assistive technology that targets limited EF capacities in adolescents (e.g., poor working memory/multitask capacity and poor inhibition of distraction), and individual differences in these functions, could reduce the high number of crashes in teen drivers. In addition, EF screening and training, video-gaming and driving simulator training, and advanced vehicle technology that collaborates with the states and traits of the driver represent potential avenues for the development of novel interventions within the field of driving.

## Figures and Tables

**Table 1:** Summary of research papers included in this review.

Author	Sample	EF measure	Driving Outcome (and Metrics)	Summary of Main Finding(s)
**Morris et al., 2008** **[45]**	i) n = 92, age: 17–25 ii) n = 244, age: 18–58	Self-Report *Global EF:* BRIEF-A	Self-Report DBQ (lapses, errors and violations)	Lower scores on all EF subscales related to more negative driving outcomes. Poor global EF explained 27% and 17% of the variance in negative driving behavior for each respective group. In addition, EF partially mediated the effects of age on negative driving behavior.
**Mäntylä et al., 2009** **[49]**	n = 50, age: 15–19	Performance *Working Memory:* N-back task Matrix monitoring *Inhibition:* Stroop task (*Interference*) Stop Signal task (*Response*) *Set-shifting:* Plus 3/Minus 3 task; Trail-Making Test	Performance Driving Simulator Lane change task (lane position and deviation)	Individual differences in EF were related to lane position and variability, but only poor working memory (not set-shifting or inhibition) significantly predicted greater variability. This effect was also mediated by computer gaming skills.
**Jongen et al., 2011** **[43]**	i) n = 31, age: 17–18 ii) n = 22, age: 22–24	Performance *Response Inhibition:* Stop Signal task	Performance Driving Simulator Task1 of normal driving and Task2 of risk-reward driving (lane position, speeding, running red lights, crashes)	Inhibitory control increased with age (suggesting continuing development), and lower inhibitory control was related to more variability in lane position, but not with risky driving behavior (speeding and red-light running).
**Roca et al., 2013 [46]**	n = 104, M_age_: 21	Performance *Cognitive Failures:* Cognitive Failures Questionnaire *Attention & Vigilance:* ANTI-V (tonic alertness/vigilance, executive control, orienting and phasic alertness indices)	Self-Report DBQ (lapses, errors, violations and aggressive driving)	The more cognitive failures reported, the higher the aberrant driving scores on the DBQ. More lapses while driving were related to more cognitive failures and poorer vigilance. No other driving behavior factors correlated with the ANTI-V. Driving errors and violations were also highly correlated with cognitive failures.
**O’Brien et al., 2013** **[37]**	i) n = 30, age: 17–21 (Speeding offenders) ii) n = 40, age: 17–21 (Controls)	Performance *Response Inhibition:* Go/No-Go task; Stop Signal task	Performance History of speeding offense	Police-reported speeding offenders had poorer inhibitory control on one performance task only (the Go/No-Go), compared to a non-offender control group.
**Graefe et al., 2013** **[53]**	n = 49, M_age_: 20.25	Performance *Interference Inhibition:* Stroop task *Set-shifting:* Wisconsin Card Sorting task; *WM, Scanning, Processing Speed:* Symbol Digit Modalities Test *Attention:* ANT (alerting, orienting, and executive attention) *Risk propensity:* BART	Performance Driving Simulator baseline task and risky driving task with time pressure and both rewards and punishments for performance (speed, lane position, stopping behavior, reaction times to hazards situations, risky overtakes)	Executive function performances did not significantly predict driving performance on the risky driving task.
**Ross et al., 2014** **[38]**	n = 46, age: 17–25	Performance *Working Memory:* Visuospatial span; Verbal Letter span *WM Load Task*: Verbal N-back task	Performance Driving Simulator lane change task at baseline and with a secondary WM task (correct lane changes, lane change initiation and path deviation)	Driving performance deteriorated overall with increasing verbal WM load on the secondary task, but drivers with better verbal WM capacity at baseline had better lane change initiation and percentage of correct lane changes. These variables were not vulnerable to the secondary task load.
**Cascio et al., 2014** **[52]**	n = 42, age: 16–17 (all male)	Performance *Response Inhibition:* Go/No-Go task	Performance Driving Simulator with either a risk promoting or non-risk promoting peer passenger (red-light running)	Higher inhibitory control related to less red-light running, but only in the presence of a cautious peer passenger.
**Ross et al., 2015 [47]**	n = 38, age: 17–25 (M_age_: 19.03)	Performance *Working Memory:* Digit span; Visuospatial span *Response Inhibition:* Stop Signal task; Go/No-Go task	Performance Driving Simulator (lane position, speeding, responses to red and yellow traffic lights, responses to road hazards, and following distance to slow vehicles)	Poor verbal working memory and inhibitory control (on the Stop Signal task alone) predicted more variability in lane position. However, poor inhibitory control alone predicted more collisions and poorer hazard detection and response. Higher visuospatial working memory performance predicted more red and yellow light running.
**Guinosso et al., 2016** **[48]**	n = 74, age: 16–24 (M_age_: 19.8)	Performance *Interference Inhibition:* Stroop task *Set-shifting:* Wisconsin Card Sort Test-64 *Attention:* Attention Network Task (ANT: alerting, orienting, and executive attention) *WM Load Task*: Verbal WM task	Performance Driving Simulator task at baseline and with a secondary WM task (velocity, accelerator position, lane position, steering wheel position)	Better Stroop inhibition and alerting predicted more consistent driving at baseline, and greater inhibitory control also predicted less variability in driving during distraction (WM load task). Flexibility, orienting, and conflict executive control were not associated with performance in either driving condition.
**Starkey et al., 2016** **[39]**	i) n = 46, age: 16–18 ii) n = 32, age: 25+	Performance *Working Memory:* Digits Forwards/ Backwards *Interference Inhibition:* Color Word Interference Test *Forward Planning:* Tower Test *Attention:* Letter Cancellation *Information Processing:* Trail-Making Test	Self-Report (a) Driving history questionnaire (b) Driver Risk Taking questionnaire (c) Driver Attitude Questionnaire	Adolescent drivers had poorer EF and were more accepting of risk. Working memory and attitudes to risk explained self-reported driving behavior, with better working memory related to more self-reported risky driving behavior and acceptance of risk. Safer driving correlated with better forward planning and less acceptance of risk.
**Ross et al., 2016** **[51]**	i) n = 30, age: 17–18 ii) n = 20, age: 22–24	Performance *Response Inhibition:* Stop Signal task	Performance Driving Simulator Task1 of normal driving and Task2 of driving with peer presence (lane position, speeding, running red and amber lights, braking and deceleration for hazards, and crashes)	Drivers with low inhibitory control showed increased speeding in the presence of peer passengers. Inhibitory control did not relate to lane position, running traffic lights, braking, or deceleration for road hazards and collisions.
**Pope, et al., 2016** **[31]**	n = 46, age: 16–19	Self-Report *Global EF:* BRIEF-SR Performance *Working Memory:* Backwards Digit Span *Set-shifting:* Trail-Making Test	Self-Report Problematic driving outcomes (crashes, citations, being pulled over)	Poor self-reported planning and organization correlated with more reports of prior crashes, with poor self-reported inhibitory control associated with prior traffic citations. Multiple BRIEF subscales had a negative correlation with being pulled over. However, there was no relationship between performance based EF measures and driving outcomes.
**Pope, et al., 2017** **[14]**	i) n = 13, age: 19–20 ii) n = 21, age: 36–54 iii) n = 25, age: 65–92	Self-Report *Global EF:* BRIEF-A	Self-Report Distracted driving behavior questionnaire	Younger and middle aged adults engaged in more distracted driving than older adults. Lower EF scores was a unique predictor of more self-reported engagement in distracted driving in all age groups.
**Hayashi et al., 2017** **[16]**	i) n = 20, M_age_: 19 (Texters) ii) n = 20, M_age_: 18.7 (Controls)	Self-Report *Global EF:* Executive Function Index	Self-Report Texting while driving questionnaire	The levels of EF on all subscales were higher in the “non-texter” (while driving) group, where “texters” had lower scores on EF subscales of strategic planning and impulse control, and lower total EF scores.
**Hatfield et al., 2017** **[50]**	n = 71, M_age_: 18.96	Performance *Response Inhibition:* Go/No-Go task Stroop task	Performance (a) Driving simulator (percentage of distance speeding and lane position) (b) Reward Saccade Task (c) Hazard Perception Task	Poor inhibitory control on the Go/No-Go alone positively correlated with total “unsafe” driving (defined unsafe responses to events), speeding in the slow zone, and overall speeding (with a large effect size).

Note: n = number of experiment participants, M_age_ = Mean age, WM = Working Memory, BRIEF = Behavior Rating Inventory of Executive Function, DBQ = Driver Behavior Questionnaire, ANT = Attention Network Test, ANTI-V = Attention Network Test for Interactions and Vigilance, BART = Balloon Analogue Risk Task.
